# Association of central venous pressure with intra-abdominal pressure, mean airway pressure and hemodynamics: an observational study

**DOI:** 10.1186/2197-425X-3-S1-A599

**Published:** 2015-10-01

**Authors:** W Huber, B Ritzer, T Lenz, M Beitz, T Lahmer, M Messer, R Schmid, B Henschel

**Affiliations:** Klinikum rechts der Isar, II. Medizinische Klinik, Munich, Germany

## Introduction

Central venous pressure (CVP) is frequently used to guide fluid therapy, although there are confounders of CVP like intra-abdominal pressure (IAP) and mechanical ventilation. Despite a large number of studies on the interactions of intra-abdominal pressure, mechanical ventilation and CVP there are few studies investigating the impact of both major confounders of CVP in the same setting.

## Objectives

Therefore, it was the aim of our study to investigate the association of CVP to IAP, mean airway pressure mP_aw_ and hemodynamic parameters derived from transpulmonary thermodilution (TPTD) in a mixed group of ICU-patients in part mechanically ventilated and in part with increased IAP.

## Methods

We analyzed a prospectively maintained hemodynamic database including 399 TPTD-measurements in 42 patients with PICCO-monitoring (Pulsion Medical Systems, Feldkirchen, Germany) and simultaneous measurements of IAP. Statistics: Spearman correlation, Wilcoxon-test for unpaired samples; multiple regression analysis; IBM SPSS 22.

## Results

Age 68 ± 12 years, 19 female, 23 male, APACHE-II 19 ± 8, SOFA 8 ± 4. IAP was increased (≥12 mmHg) in 162/399 (40.4%) measurements, and patients were under mechanical ventilation in 262/399 (65.7%) of measurements. 20.6% of measurements were without ventilation and without increased IAP, 26.6% with both ventilation and increased IAP. CVP significantly correlated to IAP (r = 0.215; p < 0.001), mP_aw_ (r = 0.196; p < 0.001) and extravascular lung water (EVLWI; r = 0.262; p < 0.001). Patients with increased IAP had significantly higher CVP (16 ± 7 vs. 13 ± 7 mmHg; p < 0.001), but comparable GEDVI (820 ± 191 vs. 799 ± 152 ml/m²; p = 0.238) versus patients with normal IAP. Furthermore, patients with mechanical ventilation had a higher CVP (15 ± 6 vs. 13 ± 7 mmHg; p = 0.001), but comparable GEDVI (796 ± 163 vs. 825 ± 175 ml/m²; p = 0.632) versus patients without mechanical ventilation. CVP was independently associated to mP_aw_ (p < 0.001; T = 4.407), to IAP (p < 0.001; T = 4.157) and to GEDVI (p = 0.035; T = 2.120), but neither to EVLWI nor to Cardiac Index CI. Changes in CVP (Delta-CVP) were independently associated to Delta-GEDVI (p = 0.044; T = 2.03) and - with borderline significance - to Delta-mP_aw_ (p = 0.066; T = 1.857), but not to Delta-IAP, Delta-EVLWI or Delta-CI. Delta-CI was independently associated to Delta-GEDVI (p < 0.001; T = 8.915) and Delta-IAP (p = 0.046; T = 2.003), but not to changes in CVP, EVLWI or mP_aw_.

## Conclusions

1. In a mixed group of patients with and without increased IAP and with or without mechanical ventilation, CVP was independently associated to IAP, mP_aw_ and GEDVI.

2. Changes in CVP were independently associated to changes in GEDVI and in mP_aw_.

3. Changes in CI were independently associated to changes in GEDVI and in IAP, but not in CVP.

4. Regarding substantial dependence of CVP from various hemodynamic and respiratory parameters, interpretation of absolute values and also changes of CVP remains to be difficult.

